# Young Children’s Affective Responses to Another’s Distress: Dynamic and Physiological Features

**DOI:** 10.1371/journal.pone.0121735

**Published:** 2015-04-13

**Authors:** Elian Fink, James A. J. Heathers, Marc de Rosnay

**Affiliations:** 1 School of Psychology, The University of Sydney, Sydney, Australia; 2 School of Education, University of Wollongong, Wollongong, Australia; University of Bologna, ITALY

## Abstract

Two descriptive studies set out a new approach for exploring the dynamic features of children’s affective responses (sadness and interest-worry) to another’s distress. In two samples (*N*_study1_ = 75; *N*_study2_ = 114), Kindergarten children were shown a video-vignette depicting another child in distress and the temporal pattern of spontaneous expressions were examined across the unfolding vignette. Results showed, in both study 1 and 2, that sadness and interest-worry had distinct patterns of elicitation across the events of the vignette narrative and there was little co-occurrence of these affects within a given child. Temporal heart rate changes (study 2) were closely aligned to the events of the vignette and, furthermore, affective responses corresponded to distinctive physiological response profiles. The implications of distinct temporal patterns of elicitation for the meaning of sadness and interest-worry are discussed within the framework of emotion regulation and empathy.

## Introduction

In early childhood affective responses to another’s distress, in particular sadness or worry, are often taken as evidence of emotional competence or maladjustment [1–3]. It is not clear, however, what these context specific patterns of affect reveal about the child. In the two studies presented here we adopt a novel approach to explore dynamic changes in children’s affective expressions during exposure to another’s distress. Temporal unfolding of affect across events—*affective chronometry*—has recently received increased attention as an object of empirical investigation [4,5]. Establishing systematic relations between affect and events unfolding in time would help to clarify the meaning of such behavior, the focus of study 1. In study 2, using a new sample of children, we utilize a physiological index of arousal to further explore temporal changes in affective responding to events, and we examine if patterns of physiological responses across events are characteristic of different affective responses. Together, these two descriptive studies enhance understanding of children’s emotional responsiveness and introduce a novel methodological approach to examine dynamic features of children’s affect.

Affective chronometry may include an examination of the *relative onset*, *peak*, and/or *recovery time* of affects in relation to circumstances or events, and across individuals [4,6]. In the adult literature, temporal changes in affect have often been studied over long time periods, sometime months [7]. However, brief time intervals have also started to receive attention and recent studies have shown that individual differences in dynamic features of affect are related to meaningful differences in personality and emotional competence [6–8]. Further, different indices of affective experience can be simultaneously analysed in a temporal manner. Thus, it is possible to ask whether multiple emotion-relevant responses—subjective feelings, expressions, and physiological changes—are related coherently over time [8,9,10]. Understanding the time-course of expressed affect in conjunction with physiological changes may elucidate the meaning of individual responses to events.

In contrast to the adult literature examining affective chronometry, research examining children’s responses to another’s distress emphasize dominant affective responses (e.g., sadness) averaged across an unfolding series of events, which typically include a depiction of distress embedded within a narrative. Children’s expressions measured in this way can then be investigated in relation to other indices of emotional competence, social engagement, or maladjustment [1,12–14]. For example, expressions of predominant sadness or worry have been associated with independent measures of children’s empathy [3,15,16]. While this approach may shed some light on the social correlates of specific affective expressions, averaging expressions across different events necessarily obscures event-specific patterns of affect elicitation and relations between different affective response modes [10]. In the child literature, it is common to elicit affective responses using a video-vignette paradigm [3,17,18] but little use is made of the different events depicted therein.

In the current study, instead of exploring associations between different affective responses and children’s social behaviour in order to understand the meaning of different affects, we utilize the affective chronometry approach to unpack their meaning. That is, we endeavour to understand the meaning of different expressions based on their dynamic temporal associations to unfolding events in carefully chosen vignettes depicting others’ distress.

Although affective chronometry has been poorly utilized in the literature in order to examine the meaning of affective responses in childhood, the comparison of affective responses across specific *event-based epochs* has provided valuable information about emotional experience and emotion regulation in infancy [19–21]. Further, physiological changes across specific event-based epochs have been examined in both infancy and childhood [11,14,18,21–25]. Temporal changes in heart rate (HR) have been linked to different patterns of information processing [14,26]: whereas HR deceleration during an emotionally challenging event is associated with the orientation of attention to the environment [22,27], HR acceleration is associated with anxiety and self-focused regulation during stressful situations [18,26]. Despite some inconsistencies in the literature [24], both infant and child research has broadly demonstrated that physiological responses across an unfolding event are meaningfully related to the event content [18,20,22,25]. Additionally, there has been some recent research that considers both the temporal unfolding of HR and affective responses in the same context. For example, Marsh and colleagues [11] examined the association between 4-year-old children’s sympathetic and parasympathetic activity and sad facial expressions during an emotion-eliciting video vignette and found that the degree of correspondence between facial expressions and physiological responses was predictive of psychopathology [23].

### Summary

The two descriptive studies presented here utilize an affective chronometry methodology to develop a novel approach to studying affective process in young children. As noted, the extant literature has focused on understanding the meaning of affective responses by examining their social correlates, as such there has been very little consideration of the *specific events* in an unfolding narrative that precipitate different affective responses or the *relative onset* of different affective reactions in relation to the narrative. However, the temporal dynamics of children’s affective responses can provide important information on the meaning of these responses in their own right. Furthermore, in examining the pattern of elicitation of children’s affective responses the extent to which different affective responses *co-occur* within an individual can also help unpack the characteristic nature of different response profiles.

Study 1, presents an exploratory examination of these features of children’s affective responding to a video vignette depicting a distressed infant in a separation scenario. Study 2 extends the findings of study 1 by varying the context in which affects are likely to be experienced and includes an index of HR change over time to better understand the temporal dynamics of children’s specific affects in response to another’s distress. Together, these two studies make an important contribution to our understanding of the meaning of children’s affective responses to another’s distress.

## Study One

There has been a small but influential literature on children’s affective displays during another’s distress at end of preschool and during the transition to school period [1,3,18]. When children are exposed to another’s distress, various important behavioral reactions can be observed (e.g., attention regulation, self-soothing behaviors). Despite the heterogeneity in this literature, most studies have demonstrated that two affective expressions—sadness and interest-worry—predominate across a variety of different stimuli [3,13,16]. These include: story vignettes, feigned emotional behaviors (by a confederate or parent), movie-scenes, cartoons, and video-vignettes of real people experiencing real emotions [10].

In the current study we were interested in the temporal pattern of affects across unfolding events in response to a commonplace theme. Parental separation was thus chosen so that the content of the vignette would be readily comprehensible for young children. The vignette depicted a real mother-infant separation in which the infant protagonist experiences distress at being left alone. This mother-infant separation vignette represents a scenario that children have likely both experienced themselves and witnessed in others. Furthermore, parental separations are known to be upsetting for young children and have been used broadly to induce affective responses [28,29]. We privileged video footage depicting authentic rather than acted scenarios taken from television or film in the hope that genuine emotion displays would resonate strongly with children and provoke clear affective responses. By focusing on a commonplace parental separation scenario, we were able to establish how children’s affective responses were associated with the anticipation, onset, and continuation of the protagonist’s distress.

To examine the temporal dynamics of children’s affective responses when faced with another’s distress, the events of the story were pragmatically divided into three epochs to reflect important aspects of the narrative: infant and mother together (*neutral*), mother initiates departure (*transitional*), and infant alone (*separation*). The thematic division of the narrative meant that affects could be examined directly in relation to contextually salient story events. On the basis of previous research [3,14,16,30], we expected to observe sadness and interest-worry in a large number of children, particularly in response to the protagonist’s distress. We also sought to determine whether these affects co-occurred in the same child, and the nature of the temporal relation between them. Our approach was descriptive and exploratory.

### Methods

#### Ethics Statement

This study was reviewed and approved by the Oxford Participant Research Ethics Committee (now known as the Central University Research Ethics Committee). Informed written consent was obtained from mothers prior to their child’s participation in the study. All children also gave verbal assent to taking part in this project.

#### Participants

Participants were 75 children (38 boys) between 54 and 72 months of age (*M*_age_ = 60 months, *SD* = 4.1 months) from a mixture of middle and working class families. All had English as a first language and no history of serious behavioral or emotional problems. Fifty-five percent of children had at least one parent who had completed tertiary education, while 41% of children had at least one parent who had completed high school or vocational training. Two children were omitted; one for non-compliance and one for compromised recording.

#### Measures and procedure

Children viewed a video of a real mother-infant separation depicting the infant’s (Tom) emotional responses—including distressed fussing and crying—in the context of a clearly defined narrative. The vignette was divided into three epochs based on the story events, resulting in epochs of similar but unequal lengths (neutral, transitional and separation; see story details in S1 Appendix). Furthermore, to ensure all children understood the vignette content, the experimenter recapitulated the story events, which resulted in epochs of slightly different lengths for each child. The neutral epoch introduces Tom and his mother (M_duration_ = 35 seconds, SD = 3.0). In the transitional epoch, Tom notices his mother’s departure but does not show marked distress (M_duration_ = 20 seconds, SD = 3.5). The separation epoch displays Tom’s sustained distress (*M*_duration_ = 28 seconds, *SD* = 3.7). To compare affective responses between epochs of unequal length, durations of sadness and interest-worry were converted to percentages for each epoch.

Children were seated next to the experimenter and discretely videotaped. Testing took place in children’s homes or in a private room at their local medical practice by appointment.

#### Continuous affective responding

Affective responses were coded continuously for the entire vignette using the Observer XT [31]. The facial coding scheme used the criteria set out by Ekman and Friesen [32] in the Facial Affect Coding System (FACS), with the inclusion of interest-worry [1,22]. The FACS is a widely used index of facial expressions whereby coders are required to learn individual muscle actions that comprise distinct affective responses. Anger, fear and joy, although coded, were virtually unobserved and are not discussed further. Sadness and interest-worry were coded as follows:

Sadness: Inner corner of brow raised; cheeks are lowered, or raised with squinted eyes; lip corners drawn down, bottom lip may be pushed outwards, including ‘cry face’, [19]. Sadness included postural sadness [33,34], characterised by a postural droop, such as a slump of the head and shoulders and often accompanied by passive downcast mouth and blank gaze. Typically, there was depressed movement but without stilling or freezing.

Interest-worry: Brows are furrowed; eyes appear wide or may be squinted with raised cheeks; can include both scanning and focused eyes; mouth may be open [1,3]. This has previously been coded as concerned-attention [35,36] and interest [37]. When this expression has been coded previously [1,22], it has sometimes been interpreted as an attempt by the child to inhibit negative emotion expressions, and therefore construed as a “modulated” expression. In the current study the majority of children exhibiting interest-worry did so intensely and there was no prima facie reason to regard the expression as a modulation of sadness or anger.

A single coder, blind to the experimental hypotheses, scored the entire sample. A second blind coder scored a random sample of 10 children (14% of the sample). Inter-coder reliability code was high: Cohen’s κs for sadness = .87 and interest-worry = .79, comparable to similar measures in the literature [28,38]. Discrepancies were resolved via discussion.

#### Affect categorization

Following Cole et al. [1], children were independently allocated into one of three groups based on their dominant expressed affect; which was not based on continuous affect coding. Classifications were: *inexpressive*, children who did not show sadness or interest-worry; *sad*, children predominantly expressing sadness; and *interest-worry*, children predominantly expressing interest-worry. While Cole et al. categorised children expressing fear and anger together with sadness, minimal fear or anger were observed in this sample. To be classified sad or interest-worry, rather than inexpressive, children needed to display the relevant emotion for more than 2 seconds across the whole vignette so that transitory expressions were not determining affect categorisation.

To calculate reliability, coders categorized 20 children (27%) based on dominant affective response during the vignette. Overall, there were two disagreements, κ = 0.85; one was between inexpressive and sad, and one was between inexpressive and interest-worry. In both cases, children were largely inexpressive and disagreements were resolved via conference with a third coder.

### Results

**Fig 1:**
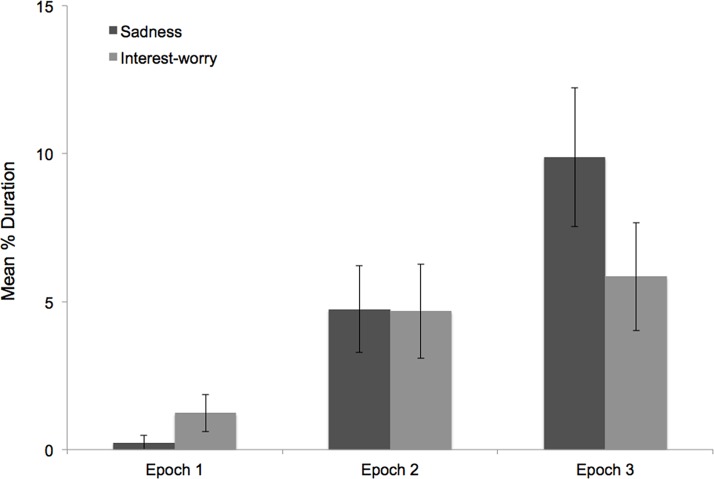
Mean Duration of Affect (Study 1). Mean percent duration of expressions of sadness and interest-worry across the three vignette epochs in Study 1 (error bars represent 1 standard error).

Fig 1 shows the mean duration of sadness and interest-worry across the three epochs (see also S1 Table). While there was very little sadness during the neutral epoch, sadness increased in the transitional epoch and escalated further in the separation epoch. An initial repeated measures ANOVA showed an effect for epoch, *F*(2, 144) = 11.79, *p* < 0.001, η_p_^2^ = 0.141. Planned contrasts showed that children expressed a greater percentage of sadness in the separation epoch compared to the transitional epoch, *F*(1, 72) = 5.40, *p* = .023, η_p_^2^ = .070. Further, the percentage of sadness was lower in the neutral epoch compared to the average of the remaining two epochs (separation + transitional), *F*(1, 72) = 22.11, *p* < 0.001, η_p_^2^ = .235. Thus, expressions of sadness were most sustained in response to the separation. This pattern of findings remained unchanged when age was controlled.

In contrast to sadness expressions, interest-worry expressions showed a marked increase between the neutral and transitional epochs but relatively little change between the transitional and separation epochs. An initial ANOVA showed an effect for epoch, *F*(2, 144) = 3.54, *p* = 0.031, η_p_^2^ = 0.047. Planned contrasts showed that there was no difference in interest-worry between the transitional and separation epochs, *F*(1, 72) = .29, *p* = .591, η_p_^2^ = .004, but the percentage of interest-worry was lower in the neutral epoch compared to the average of the remaining two epochs, *F*(1, 72) = 12.08, *p* = 0.001, η_p_^2^ = .144. This pattern of findings remained unchanged when age was controlled.

These findings suggest that interest-worry peaks prior to the protagonist’s sustained displays of distress, and remains high thereafter. Sadness, on the other hand, escalates in response to the protagonist’s distress, peaking during the separation epoch. Given the different temporal patterns of elicitation for these two affects, we sought to determine to what extent they co-occurred within the same individual.

Based on dominant affect categorisations, there were in 31 inexpressive (42%; 16 girls), 24 sad (33%; 10 girls), and 18 interest-worry children (25%; nine girls). Although the protagonist of the separation vignette was male, affect classification was unrelated to sex, χ^2^_2_ (*N* = 73) = .58, *p* = .750. There was no significant difference in age across the three affect categorisations, *F*(2, 70) = 1.22, *p* = 0.302.

**Fig 2:**
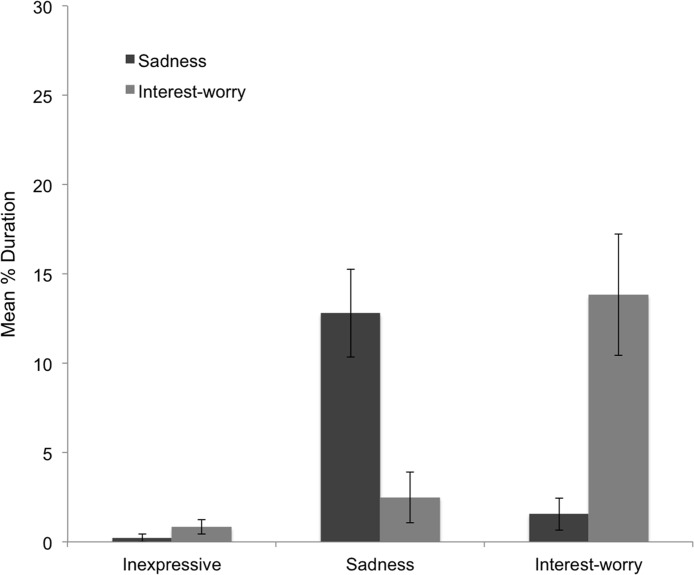
Duration of Affect by Affect Group (Study 1). Mean duration of sadness and interest-worry as a function of affect group in the mother-infant separation vignette (error bars represent 1 standard error).

Two children classified as inexpressive displayed sadness for less than 2 seconds, all children expressing any interest-worry did so for more than 2 seconds. Fig 2 shows the extent to which dominant affect classification overlapped with independently scored continuous affect. While affect groups *necessarily* co-vary with continuous scores, Fig 2 is striking in that children classified sad showed very little interest-worry (though 7 children did show some), and those classified interest-worry showed very little sadness (only 3 children showed any). Correlations between expressions of sadness and interest-worry across each of the three epochs confirmed that there was no association across affective responses, *r*(73)s between -.041 and. 085, *p*s >.477. Nor was there a significant correlation between duration of sadness and interest-worry overall across the whole vignette, *r*(73) = -.022, *p* = .856. Although it should be noted that given the small number of children displaying both sadness and interest-worry, these correlations should be interpreted cautiously. Together, the independent affect coding systems indicated that there are divergent responses to the vignette, suggesting qualitatively distinct affective profiles.

### Discussion

In study 1, children’s affective responses to another’s distress were examined using a novel approach, by focusing on the temporal unfolding of expressive behavior. There were several notable findings. First, expressions of sadness and interest-worry showed distinct temporal associations with the events of the vignette. Whereas duration of sadness increased steadily in response to the protagonist’s expressed distress, interest-worry had an earlier peak, which was prior to the protagonist’s sustained distress. Second, categorization on the basis of dominant affect strongly distinguished children: when children were affectively expressive either sadness or interest-worry clearly predominated, and most children expressed one affect exclusively across the course of the vignette. The relative independence of sadness and interest-worry, in conjunction with their different sequence of elicitation, together suggest that sadness and interest-worry reflect distinctive response modes to another’s expressed distress.

However, the vignette used in study 1 presents some methodological issues that make interpreting the meaning of these different affective responses difficult. The epochs were of unequal length and although this was accounted for by correcting to percentage duration, greater precision in the epoch length would allow more systematic analyses of the time-course of affective expressions. Furthermore, unsystematic variation was introduced by the experimenter's recapitulation of the major story events in the neutral and transitional epochs.

Further, the current findings do not clarify whether children were responding to specific story events or whether they were merely responding contagiously. We think this latter possibility unlikely because the context was a commonplace event that should not be emotionally overwhelming for most children, and those children who were expressive responded with a moderate amount of sadness and interest-worry rather than prolonged periods of distress (as is depicted by the protagonist). Additionally, when sadness and interest-worry were expressed, these affects showed differential relations to events. Nevertheless, given the temporal correspondence between expressions of sadness and the protagonist’s distress in particular, a contagious mechanism needs further investigation. It is important to ask whether the elicited levels of sadness and interest-worry were due to children’s understanding of the protagonist’s predicament or whether they simply mirror of the protagonist’s emotional experience. If the observed affective responses are best conceived as a contagious response there is no reason to expect that the context or narrative is of particular importance and, therefore, we would expect similar levels of affect in both contextualised and decontextualized situations. Thus, in study 2, we first presented children with an infant expressing similar levels of distress to Tom (study 1), but in a decontextualized setting.

Despite these methodological concerns, study 1 starkly revealed that children respond in very distinctive ways to a commonplace event depicting another’s distress. Further, there was some suggestion that affective responses were meaningfully associated with specific story events. In study 2, we build on these foundations and introduce a physiological criterion against which to explore the meaning of the specific temporal patterns of different affects.

## Study 2

Study 2 was designed to clarify and extend the findings presented in study 1 with a new sample of similarly aged children. A larger sample of children was sought because of the individual differences in expressions of sadness and interest-worry, observed in only 33% and 25% of children respectively. The first aim of study 2 was to establish whether children would express affect when faced with a crying infant (*Distressed Infant*; vignette 1),but without any contextual information (i.e., a narrative) with which to make sense of the infant’s predicament. The Distressed Infant vignette serves as a comparison to the mother-infant separation vignette presented in study 1 (specifically the separation epoch where the infant displays sustained distress).

The second aim of study 2 was to examine the temporal pattern and co-occurrence of sadness and interest-worry expressions during a different vignette depicting another’s distress. Thus, children were presented with a new parent-child separation vignette, this time depicting a similar aged child’s first day of school (*First Day*; vignette 3). The rationale for selecting the First Day vignette follows study 1: it depicts a real child involved in an emotional separation from his parents (see Methods). The First Day vignette also allowed us to track the relative onset of affects with greater acuity when compared to study 1 as it could be divided into nine short epochs of equal time (10 seconds), and did not require explanation. In this manner, the dynamics of affective responses could be more accurately analysed over time.

The final aim of study 2 was to examine phasic changes in children’s HR in order to unpack the dynamic nature of children’s affective responses to another’s distress. As phasic changes in HR are temporally sensitive and linked to specific events, it is typical to measure HR changes during a pre-determined *critical period* [14,22,27]. In the current study, the critical period was determined a priori to be the most emotionally intense section of the First Day vignette, when the protagonist displays clear prolonged distress at being left at school by his parents. To provide a contrast, changes in children’s HR were also examined during the first three epochs of the First Day vignette, during which there is foreboding of later distress but the protagonist does not display clear prolonged distress.

In sum, the current study adopted a novel descriptive approach to further explore dynamic features of sadness and interest-worry expressions in response to another’s distress. First, to distinguish between two possible interpretations for children’s affective responses, in study 2 children were initially presented with a vignette depicting a distressed infant in the absence of a narrative similar to that presented in the separation epoch of the mother-infant separation vignette (study 1). If the pattern of affective responses found in study 1 were due to emotion contagion, then we would expect to see similar affective responses to the Distressed Infant vignette in study 2. However, if affective expressions are in fact a response to the distressed child’s predicament, which has to be understood in a context, then the decontextualized nature of the Distressed Infant vignette will make it hard for children to understand the situation from the protagonist’s point of view, and we would expect relatively little affect. Second, using a new separation vignette (First Day) with a new sample of young children, it was expected that the temporal pattern of elicitation and co-occurrence of sadness and interest-worry would somewhat replicate that found in study 1. Thus, we expected that the onset and peak duration of interest-worry would precede a full realisation of the protagonist’s predicament, whereas sadness would continue to escalate during the critical period of emotional intensity in response to the protagonist’s worsening condition.

Finally, it was expected that HR changes would be meaningfully related to the unfolding events of the First Day vignette: given the vividness of this vignette, we expected an initial HR response indicative of attention capture (deceleration), with HR acceleration during the critical period of the protagonist’s distress. As temporal changes in children’s physiological responding across an event have sometimes been shown to be associated with specific affective responses [11,14,18,23], we examined if phasic changes in HR were characteristic of different affects.

### Methods

#### Ethics Statement

We obtained approval to conduct this study from the Human Research Ethics Committee of the University of Sydney, Australia. All parents provided written informed consent prior to their child’s participation in the study. All children also provided verbal assent before taking part in the project.

#### Participants

Participants were 114 kindergarten children (58 boys) between 55 and 77 months (*M*_age_ = 67 months, *SD* = 5.0 months) recruited from three schools. There were no exclusion criteria. Children came from a mixture of ethnic backgrounds common to the area, and had English as a native language. No child had history of serious behavioral or emotional problems as reported by teachers. Fifty-eight percent of children had at least one parent who had completed tertiary education, while 40% of children had at least one parent who had completed high school or vocational training.

As is typical, not all physiological data was useable; 1 child was away from school on testing day, 3 children did not wish to complete the task with the physiological equipment, 2 children had corrupted files, 4 children had ectopic heart beats/arrhythmia, 6 children had errors to over 5% of the data points, and the signal was degraded for 4 children. Thus, the data for 94 children (48 boys) was used in HR analyses (M_age_ = 67 months, SD = 5.1 months). There was no significant difference in age between children that were included and excluded from the physiological analyses, *t*(112) = .79, *p* = .429.

#### Measures and Procedure

In a quiet room at school, children watched the Distressed Infant vignette (56 s), the Happy Infant vignette (40 s; the same infant, without any contextual information, expressing joy and laughter), and the First Day vignette (93 s) in a fixed order [1,12] on an LCD monitor. Both the Distressed Infant and First Day vignettes presented a protagonist expressing sustained distress for approximately 40 seconds. There was a 20 second gap between each vignette and we do not report children’s responses to the Happy Infant vignette (only 2 children showed any sadness and 6 showed any interest-worry). For a more detailed description of the full *emotion elicitation* procedure, see Heathers, Fink, Kuhnert, and de Rosnay [39]. All vignettes had a positive resolution. The experimenter (seated beside the child) also watched the vignettes maintaining neutral affect. Children were discretely videotaped.

The Distressed Infant and First Day vignettes were chosen because, while upsetting, they were not outside the experience of typical children, and should not cause over-arousal in well-regulated children [22]. Distressed Infant was created to show a seated infant who, from a neutral start, becomes progressively more distressed until he is finally handed his bottle and is no longer distressed. Throughout the distress it is not clear that he is hungry. First Day had several valuable features that merited its use: i) it had a clear narrative; ii) it had a resolution; iii) the protagonist was a similar age as participants; iv) the protagonist’s expressions of distress were predictable given the foreboding events; v) the protagonist’s affective and behavioral responses were clearly recognisable; vi) the vignette, an excerpt from a documentary, represented genuine emotional responses. The critical period was defined between epochs 4 and 6 when the protagonist shows clear and prolonged distress (see S1 Appendix). It was not possible to source equivalent male and female versions but analyses were conducted to examine gender differences in responding.

Heart rate was recorded via a blood volume pulse (BVP) sensor attached to a Biograph Procomp2 unit [40] placed on the index finger of the right hand. If a child objected, he/she was able to watch the vignettes without the sensor attached. To decrease movement artefacts, children were asked to keep their right hand as still as possible. At different assessment sessions, children also completed a range of tasks that are described in Fink [41].

#### Continuous affective responding

Sadness and interest-worry were coded continuously for all vignettes using the procedures from study 1. A second coder blind to the study hypotheses coded a random sample of 20 children (18%). Inter-code reliability was high, Cohen’s κ for sadness = .76, and for interest-worry = .77. (Anger, fear and joy were also coded but were rare and are not discussed further.)

#### Affect categorization

In keeping with study 1, children were independently categorized based on their dominant expressed affect during the First Day vignette. A second coder classified 50 children (44%) into affect categories to determine reliability. Agreement was excellent, κ = 0.92. Disagreements (5 codes) were resolved via conference with a third experienced coder.

#### Heart rate

Using a BVP collection method for heart rate has compared favourably with more traditional ECG methods [39,42]. As such, the BVP curve was devolved into PP (pulse-to-pulse) intervals using custom software in LabView 9.0 [40], which were used to derive heart rate period (HRP). HRP represents the period of time between successive heart beats, as such, a HRP decrease represents an *acceleration* in heart rate, while a HRP increase represents a deceleration in heart rate.

### Results

#### Specificity of affective responses

**Fig 3:**
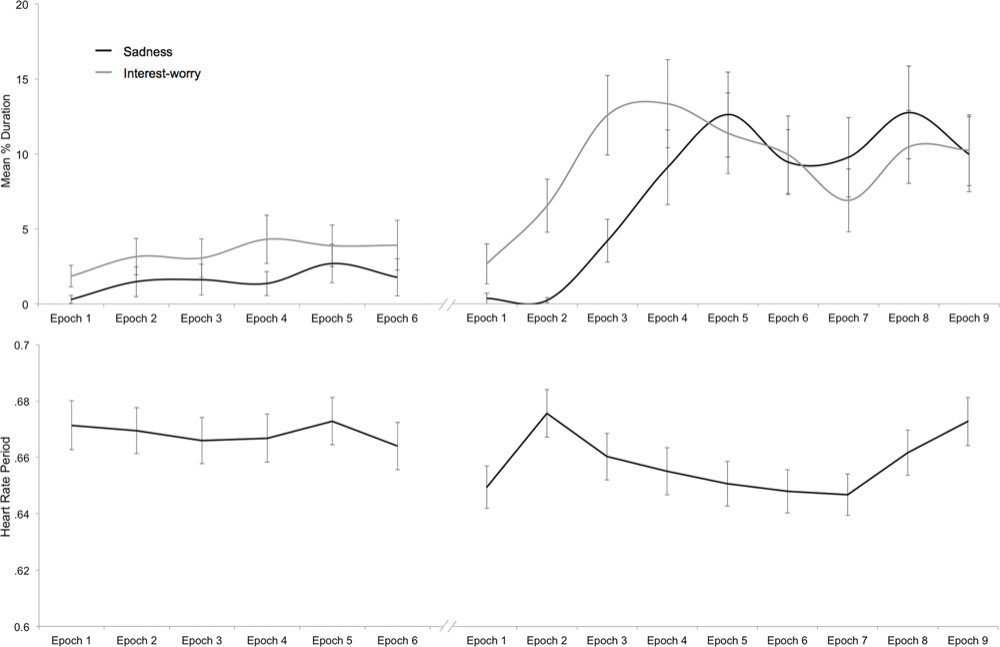
Mean Duration of Affect and Heart Rate Period (Study 2). Mean duration of expressions of sadness and interest-worry of overall sample (top) and heart rate period (bottom) during 10-second epochs of the Distressed Infant and First Day vignette. (Epoch 6 for Distressed Infant represents percent duration over 6 seconds, not 10 seconds).

Using continuous emotion scoring, children’s responses to the Distressed Infant vignette (see Fig 3) were first compared with the overall pattern of responding to the mother-infant separation vignette in study 1. Regarding sadness, only 8.0% of children expressed any sadness during the Distressed Infant vignette, compared with 39.7% during study 1, this proportional difference was significant, *z* = 2.20, *p* = 0.028. Further, a repeated measures ANOVA did not reveal a significant difference in the amount of sadness expressed by epoch during the Distressed Infant vignette, *F*(5, 560) = .81, *p* = .545, η_p_^2^ = 0.010. In contrast, expressions of sadness were profoundly associated with epoch in study 1. Regarding interest-worry, 25.7% of children expressed any interest-worry during the Distressed Infant vignette, which was more consistent with study 1 (i.e., 31.5%); this proportional difference between studies was not significant, *z* = .87, *p* = .386. However, a repeated measures ANOVA again showed that there was no significant difference in the amount of interest-worry expressed by epoch during the Distressed Infant vignette, *F*(5, 560) = .66, *p* = .651, η_p_^2^ = 0.006. In contrast, in study 1, expressions of interest-worry were robustly associated with epoch.

There were also differences in the amount of affect elicited within study 2, between the Distressed Infant and First Day vignettes (see Fig 3 and S2 Table). Regarding sadness, 31% of children expressed some sadness during the First Day vignette; a McNemar test for correlated proportions shows that the proportional difference in the number of children expressing sadness in the First Day compared to Distressed Infant vignette was significant, *p* < 0.001. A repeated measures ANOVA revealed that, unlike the Distressed Infant vignette (see above), there were significant differences in the amount of sadness by epoch of the First Day vignette, *F*(8, 896) = 6.75, *p* < 0.001, η_p_^2^ = 0.057. Regarding interest-worry, 50% of children expressed interest-worry during First Day vignette; a McNemar test for correlated proportions revealed that the proportional difference in the number of children expressing interest-worry in the First Day compared to Distressed Infant vignette was significant, *p* < 0.001. A repeated measures ANOVA revealed that, unlike the Distressed Infant vignette (see above), there were significant differences in the amount of interest-worry by epochs of the First Day vignette, *F*(8, 896) = 3.23, *p* = 0.001, η_p_^2^ = 0.028.

In sum, affective responses were more frequent during contextualised distress (study 1 and First Day, study 2), when compared to decontextualized distress, particularly for expressions of sadness. Furthermore, the pattern of sadness and interest-worry differed across epoch for contextualised distress only. While these comparisons were not experimental, Figs 1 and 3 reveal marked differences in children’s responses to contextualise distress.

#### Temporal pattern and co-occurrence of affective responses to First Day vignette

Fig 3 shows that First Day was very emotionally evocative but that, as in study 1, interest-worry escalated more rapidly than sadness and peaked prior to the critical period (epochs 4–6). By contrast, sadness peaked precisely in the 5^th^ epoch. There was a profound influence of epoch on both expressions of sadness and interest-worry (see above). It is also noteworthy that levels of both affects remained relatively high throughout the remaining epochs, even though the protagonist only expressed distress for the first 2 seconds of the 8^th^ epoch, and was united with his mother in the 9^th^ epoch.

Affect categorisation yielded 45 inexpressive (40%; 17 girls), 33 sad (29%; 17 girls), and 35 interest-worry (31%; 21 girls) children. All children expressing any sadness did so for more than 2 seconds. Five children classified as inexpressive displayed interest-worry for less than 2 seconds. As in study 1, affect duration, *t*_sad_(111) = .95, *p* = .345; *t*_interest-worry_(111) = 1.58, *p* = .117, and affect categorisation, χ^2^_2_(*N* = 113) = 4.04, *p* = .132, were unrelated to sex. There was no significant difference in age across the three affect categorisations, *F*(2, 110) = .985, *p* = 0.377.

**Fig 4:**
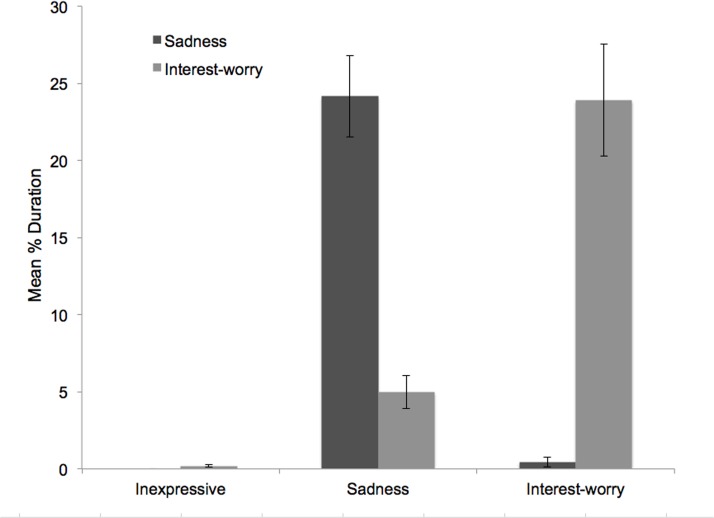
Duration of Affect by Affect Group (Study 2). Mean duration of sadness and interest-worry as a function of affect group in the First Day vignette (error bars represent 1 standard error).

**Fig 5:**
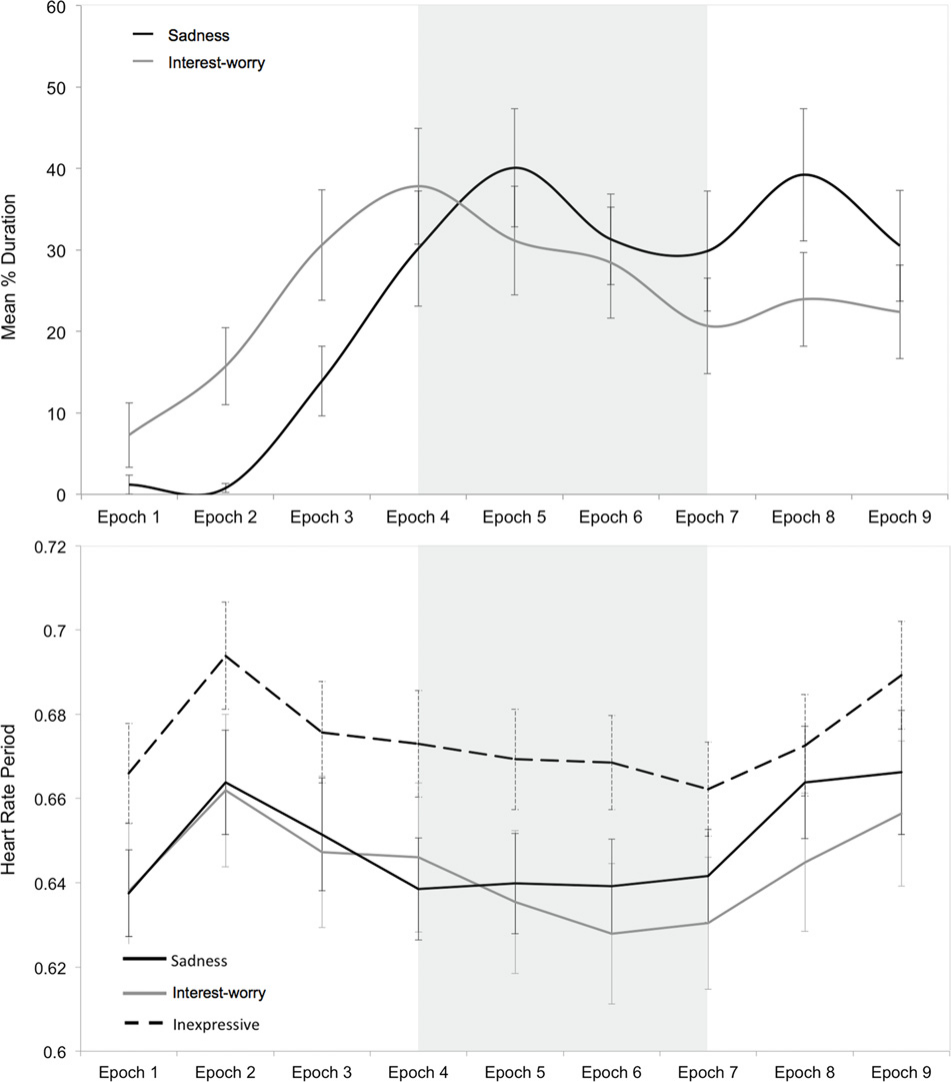
Mean Duration of Affect and Heart Rate Period (Study 2). Mean duration of expressions of sadness and interest-worry (top) and heart rate period changes as a function of affect group (bottom) during 10-second epochs of the First Day vignette. Shaded area delineates the critical period (error bars represent 1 standard error).

In keeping with study 1, Fig 4 shows that affect group categorisation resulted in distinct groups. Correlations between expressions of sadness and interest-worry across each of the nine epochs confirmed that there was no association across affective responses, *r*(113)s between-.15 and-.02, *p*s >.102. Nor was there a significant correlation between overall duration of sadness and interest-worry across the whole vignette, *r*(113) = -.08, *p* = .380. As in study 1, given the small number of children displaying both sadness and interest-worry, these correlations should be interpreted cautiously. The temporal pattern of sadness and interest-worry responses by affect group (Fig 5) broadly mirrors that of the whole sample (compare with Fig 3).

**Fig 6:**
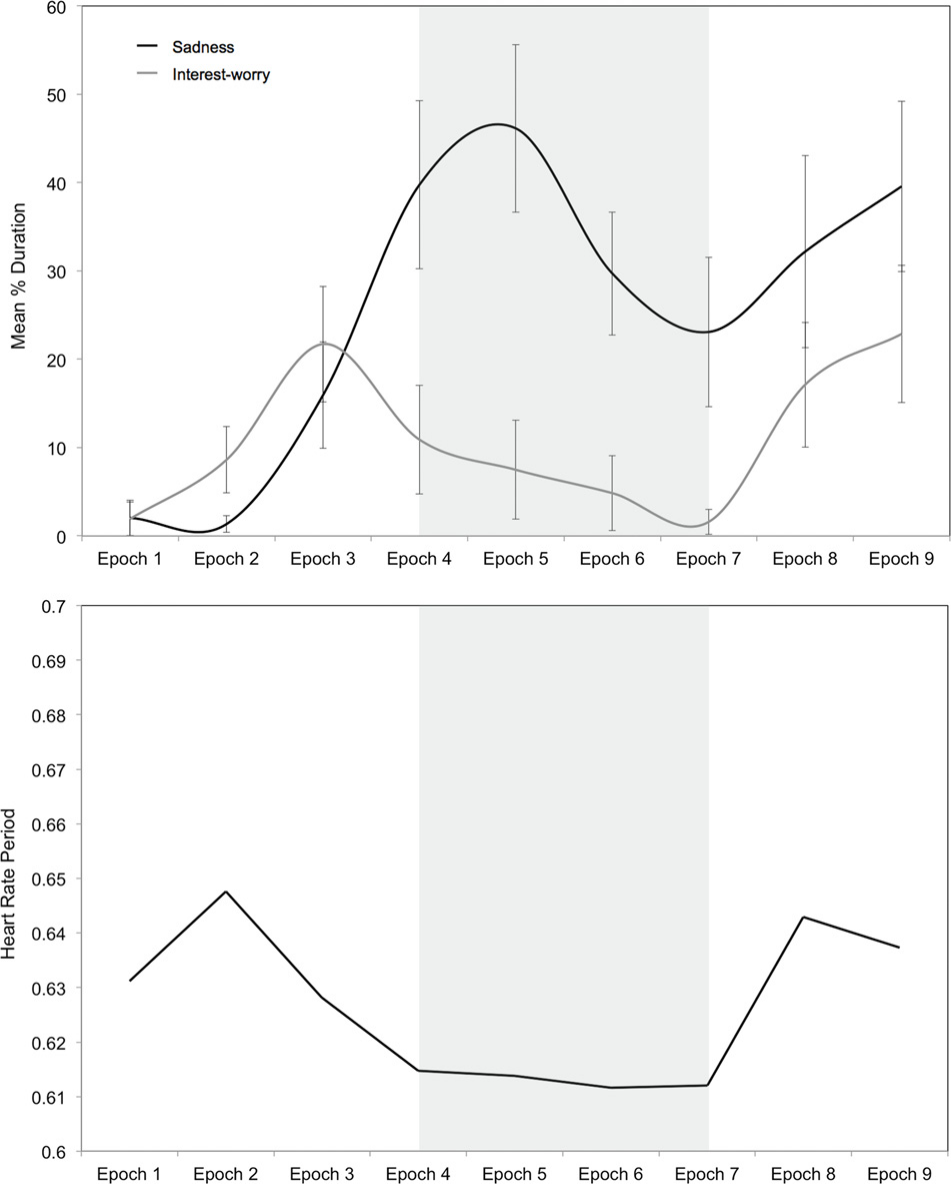
Mean Duration of Affect and Heart Rate Period (Study 2). Mean duration of expressions of sadness and interest-worry (top) and heart rate changes (bottom) during 10-second epochs of the First Day vignette for children that expressed both affects, *n =* 19. Shaded area delineates the critical period (error bars represent 1 standard error).

Notwithstanding the marked division in affect categorisation, there were some children who expressed both sadness and interest-worry. When both affects were expressed, children were more likely to be dominant-sad (17/19, 89%) than dominant-interest-worry (2/19, 11%). A McNemar test showed that this asymmetry was significant (*p* <. 001). However, the proportion of children expressing both affects did not differ between study 1 and study 2, *z* = 1.34, *p* = .165. Thus, while dominant-interest-worry children appear to be a very distinctive group, there was a subset of dominant-sad children displaying a low level of interest-worry; these latter children were clearly sad dominant in the critical period (see Fig 6).

#### Phasic HRP change

Children’s phasic HRP during the Distressed Infant vignette was relatively stable across all epochs, with no marked acceleration or deceleration (see Fig 3). A repeated measures ANOVA showed no significant difference in HRP across epochs, *F*(5, 440) = 1.71, *p* = 0.132, η_p_^2^ = 0.019. During the First Day vignette, by contrast, there were significant difference in HRP across epochs, *F*(8, 704) = 15.18, *p* < 0.001, η_p_^2^ = 0.147, and these phasic HRP changes appeared meaningfully related to the unfolding events. As predicted, there was an initial deceleration in children’s HR, suggestive of attentional capture [26], but the critical period of the vignette corresponded to modest HR acceleration (see Fig 3). At the resolution of the protagonist’s distress and his reunion with his mother (Epochs 8 and 9), children’s HR again began to decelerate.

Fig 5 shows phasic HRP changes across First Day by affect group. First, it is important to note the largely uniform pattern of HRP change across all groups. Second, there was no significant difference in HRP by group, *F*(2, 86) = 1.78, *p* = .176, despite a suggestion in Fig 5 that inexpressive children have higher overall HRP (i.e., lower HR) compared to sad and interest-worry children. This pattern of findings remained unchanged when age was included as a covariate in the model.

To examine whether distinct patterns of HRP change were characteristic of different affect group, a trend analysis was conducted to examine changes in HRP during the initial three epochs and the critical period of the vignette (see Fig 5). The number of children in each affect group was: 37 inexpressive, 27 interest-worry, and 25 sad. Trend analysis examining HRP across the first three epochs by affect group revealed significant linear, *F*(1, 86) = 5.19, *p* = .025, η_p_^2^ = .057, and quadratic, *F*(1, 86) = 63.56, *p* < 0.001, η_p_^2^ = .425, trends, suggesting that children’s HRP increased (i.e., HR deceleration) across the first three epochs, with a turning point at epoch 2 (i.e., the quadratic trend). There was no HRP by affect group interaction, however, suggesting that patterns of HRP change were similar for all affect groups during the initial three epochs, *F*s(2, 86) < 0.27, *p*s >. 766, η_p_^2^ <. 006.

Over the critical period, trend analysis contrasts revealed a significant linear trend for HRP, *F*(1, 86) = 5.82, *p* = .018, η_p_^2^ = .063, and a significant affect group x HRP interaction for the linear trend, *F*(2, 86) = 3.21, *p* = .045, η_p_^2^ = .069. Fig 5 shows that inexpressive and interest-worry children display HR acceleration during the critical period whereas there was relative stability in HR for children expressing sadness. Follow-up pairwise comparisons revealed a significant difference in the linear slope between sad and interest-worry children, *F*(1, 86) = 5.85, *p* = .018, η_p_^2^ = .064, but not between inexpressive and sad children, *F*(1, 86) = 0.53, *p* = .470, η_p_^2^ = .006, nor between inexpressive and interest-worry children *F*(1, 86) = 3.65, *p* = .059, η_p_^2^ = .041. Simple post-hoc follow-up tests were then run to see if the individual affect group slopes were significantly different from 0 (i.e., stable HRP change across the critical period). The HRP of children expressing sadness was stable during the critical period, *t*(24) = .13, *p* = .895, that is there was no acceleration or deceleration during these epochs. However, children expressing interest-worry showed a significant slope, *t*(26) = -3.63, *p* < 0.01, suggestive of HR acceleration during the critical period. Similar to children expressing sadness, children who were inexpressive has stable HRP across the critical period, *t*(36) = -.95, *p* = .356.

### Discussion

Study 2 employed a novel descriptive approach to further examine dynamic changes in children’s affective responses to another’s distress. First, a Distressed Infant comparison vignette was introduced to facilitate comparison between contextualised (mother-infant separation vignette; study 1) and decontextualized (Distressed Infant vignette; study 2) expressions of distress. Second, we examined whether the distinctive temporal patterns of sadness and interest-worry observed in study 1 would be approximately replicated in a new sample of children with a different parental separation vignette (First Day). Finally, phasic changes in HRP were introduced to further explore dynamic changes in children’s affective responses. A brief discussion of the results is presented below; the broader implications of these findings are considered in the General Discussion.

When presented with the Distressed Infant vignette, relatively few children responded affectively, furthermore, there was little change in either affect or HRP across epochs despite equivalent amounts of depicted distress. Notably, if children did respond affectively to the Distressed Infant vignette they were likely to response with interest-worry not sadness. By contrast, 60% of children responded with overt affect to the First Day vignette, and both affective responses and HRP differed as a function of epoch. If affect were merely a contagious response, we would expect to see a similar response to Distressed Infant, in which clear distress was expressed for a comparable length of time (see Methods). Of course the infant protagonist may have been less salient to child participants than the same-age child but recall in study 1 that 58% of children also expressed affect in the mother-infant separation vignette. Taken together, these comparisons support the interpretation that affective responses are contingent, at least to some extent, on children’s understanding the unfolding narrative around the expressions of a protagonist’s expressed distress, particularly for expressions of sadness. Of course these interpretations need to be confirmed using experimental, rather than exploratory, methods, but it is noteworthy that Hepach, Vaish, and Tomasello [43] have shown that young children appear reluctant to sympathise with a distressed confederate when the expression of distress seems unjustified; it seems that children’s willingness to feel or express empathy turns on their understanding of the victims circumstances.

Responses to the First Day vignette again revealed that peak duration of sadness expressions directly corresponded with the protagonist’s sustained distress. Greater temporal acuity in this study further confirmed the close correspondence between the protagonist’s distress and children’s sad affect. By contrast, expressions of interest-worry again anticipated the protagonist’s sustained distress, broadly replicating study 1. Regarding the co-occurrence of affective expressions, sadness and interest-worry were largely independent, defining different groups of children as in study 1.

Across the entire sample, there were marked and largely uniform changes in HRP during the First Day vignette that corresponded to the protagonist’s emotional state. These systematic changes in HRP were not observed during the Distressed Infant vignette, highlighting the importance of contextual understanding, not only for affective responses, but also affect-related physiological indices.

In addition to uniform changes in HRP during the First Day vignette, HRP changes in this vignette also supported the conception of sadness and interest-worry as distinct response modes. During the critical period of the First Day vignette, expressions of sadness corresponded with neither HR acceleration nor deceleration. By contrast, expressions of interest-worry corresponded with HR acceleration during the same period, which is typically associated with parasympathetic nervous system withdrawal or sympathetic nervous system activity, and indicative of threat perception [18,26].

Together, studies 1 and 2 demonstrate the distinctive temporal pattern of elicitation of children’s expressions of sadness and interest-worry to another’s contextually relevant distress and, further, these studies strongly suggest that these expressions are largely independent response modes enacted by different children. In so far as they overlap, sadness was clearly dominant. In the General Discussion, we position these findings within the broader literature on children’s emotional competence and discuss some limitations of our approach.

## General Discussion

Across two studies, distinctive temporal patterns of sadness and interest-worry expressions were observed. Whereas duration of sadness peaked in response to the protagonist’s sustained distress, expressions of interest-worry anticipated the protagonist’s sustained distress. These expressions were also found to be largely independent, with somewhat distinct patterns of physiological arousal. Having distinguished two response modes to another’s distress it is important to ask whether this differential pattern can go some way to clarifying the meaning of children’s responses, and how they may correspond to socio-emotional competence.

Understanding the temporal features of different affects may be particularly beneficial for the empathy and emotion regulation literatures [15,44,45], which have both grappled with how to interpret children’s differing affective responses to emotionally challenging situations. To date, these literatures have largely sought to validate the interpretation of affective expressions against broader measures of social behavior. By examining the onset and peak of affective expression, and associated physiological profiles, a tentative interpretation can be presented. In both study 1 and study 2, peak duration of sad affect occurred when the protagonist displayed sustained distress, a temporal pattern of elicitation consistent with the commonplace understanding of empathy as affective matching [15,46]. Thus, sad expressions may possibly be described as a manifestation of *empathic sadness*, an empathic response to the protagonists’ predicament and distress [15,45]. In contrast, in both study 1 and study 2, the onset of interest-worry was more rapid and the expression peaked prior to a full comprehension of the protagonist’s actual predicament, a temporal pattern perhaps consistent with anxious apprehension [47]. Although, it is important to note that there may be alternative interpretations of the meaning of expressions of interest-worry. For example, children expressing interest-worry may be more skilled at anticipating the unfolding events, and therefore this expression peaks prior the overt manifestation of the vignette protagonist’s emotions. However, an examination of the physiological profile of children expressing interest-worry supports the interpretation of this affective response as anxious, as these children were likely to experience HR acceleration during the critical period of the vignette; indicative of anxiety and poor regulation. Together, the pattern of affective and physiological responding of children expressing interest-worry fits the profile of *personal distress* rather than *sympathy*, as described by Eisenberg and colleagues [15,45,48]: individuals who are personally distressed experience, “… a self-focussed, aversive affective reaction to the apprehension of another’s emotion, associated with the desire to alleviate one’s own, but not the other’s distress” p. 72 [45]. Further research on the temporal dynamics of children’s affective responses to other emotionally-challenging events, in addition to a thorough examination of the patterns of recovery over time of these expressions would provide further understanding of the meaning of these affective responses in different contexts.

Despite a firm theoretical basis for expecting a distinction between different affective responses to another’s distress [15,45], it nonetheless remains challenging to explain why specific affective responses, such as sadness and interest-worry have not previously emerged as distinctive reactions to distress in the empirical literature. We think this hinges on a fuller consideration of the significance of the eliciting context [49], especially in light of the findings of study 2. In previous research a wide range of different stimuli have been utilised to elicit affective responses from children. For example, Gurthrie et al. [36] showed children a vignette about a young girl burnt in a house fire and her subsequent rehabilitation, and Eisenberg, McCreath and Ahn [2] showed children a movie depicting personal injury. It is plausible that, when faced with someone in distress resulting from injury, a sympathetic response includes aspects of facial worry and concern. In keeping with this interpretation, a recent study by Bandstra and colleagues [50] demonstrates that young children exhibit a different pattern of empathy-related behavioral responses when faced with a confederate’s injury compared to her sadness. Thus, it would be misguided to assume that all instances of sadness represent sadness for the protagonist’s distress, or that interest-worry is necessarily personal distress [22]. Hence, the interpretation of sadness and interest-worry presented here must be viewed in light of the specific context used to elicit these responses, a caveat especially pertinent given the absence of affective responses observed when children viewed a distressed infant without any contextual cues. Similarly, physiological responses are also likely to be highly dependent on the context of elicitation [51].

While examining the temporal pattern of elicitation of children’s sadness and interest-worry can help interpret the meaning of these expressions, this method is less well suited to examining the meaning of inexpressive responses. Given that a large proportion of children in both studies were inexpressive throughout the vignettes it is unlikely that this response represents insensitivity to or callousness about the unfolding events [52]. The pattern of inexpressive children’s HR changes across the whole vignette was statistically indistinguishable from their more expressive counterparts, and there was no significant difference in the pattern of HRP change of inexpressive children when compared to those expressing sadness or interest-worry during the critical period, suggesting that these children are not insensitive to the protagonist’s distress. To better understand the meaning of inexpressive responses to another’s distress, it would be helpful to simultaneously examine their behaviors in other socio-emotional domains, an issue addressed in Fink [41].

In sum, the two studies presented here draw on the affective chronometry approach to map dynamic changes in affective responses to another’s distress to better understand the meaning of different affects. By examining the temporal relation of sadness and interest-worry to unfolding vignette events, the co-occurrence of expressions and their physiological profile, a tentative interpretation of these affects is presented. To further understand the meaning of children’s affective responses, research is needed that employs both an affective chronometry approach in combination with external measures of children’s socio-emotional competence to validate interpretation.

## Supporting Information

S1 Table Mean duration of affective responses (Study 1). Mean percent duration of affective responses (standard deviations in parentheses) during the Mother-Infant Separation Vignette (study 1).
(DOCX)

S2 Table Mean duration of affective responses (Study 2). Mean percent duration of affective responses (standard deviations in parentheses) during the First Day Vignette (study 2).
(DOCX)

S1 Appendix Detailed narrative of separation vignettes. Detailed narrative of mother-infant separation vignette (study 1) and First Day vignette (study 2) by epoch
(DOCX)

S1 Data Data from Studies 1 and 2. (ZIP)
