# Supplementary material for: Young Children’s Affective Responses to Another’s Distress: Dynamic and Physiological Features
Source: PLoS One. 2015 Apr 13;10(4):e0121735. doi: 10.1371/journal.pone.0121735 (PMC4395218; doi:10.1371/journal.pone.0121735)
Supplement: S1 Table — Mean percent duration of affective responses (standard deviations in parentheses) during the Mother-Infant Separation Vignette (study 1). (DOCX) [file pone.0121735.s001.docx]

S1 Table. Mean duration of affective responses (Study 1)

Mean percent duration of affective responses (standard deviations in parentheses) during the Mother-Infant Separation Vignette (study 1).

|  | Study 1. Mother-Infant Separation Vignette  Epochs | | |
| --- | --- | --- | --- |
|  | 1 | 2 | 3 |
| Sadness  (% duration) | .23  (2.04) | 4.45  (12.53) | 9.89  (20.08) |
| Interest-worry  (% duration) | 1.24  (5.41) | 4.68  (13.57) | 5.84  (15.63) |
