# Supplementary material for: Young Children’s Affective Responses to Another’s Distress: Dynamic and Physiological Features
Source: PLoS One. 2015 Apr 13;10(4):e0121735. doi: 10.1371/journal.pone.0121735 (PMC4395218; doi:10.1371/journal.pone.0121735)
Supplement: S2 Table — Mean percent duration of affective responses (standard deviations in parentheses) during the First Day Vignette (study 2). (DOCX) [file pone.0121735.s002.docx]

S2 Table. Mean duration of affective responses (Study 2)

Mean percent duration of affective responses (standard deviations in parentheses) during the First Day Vignette (study 2).

|  | Study 2. First Day Vignette  Epochs | | | | | | | | |
| --- | --- | --- | --- | --- | --- | --- | --- | --- | --- |
|  | 1 | 2 | 3 | 4 | 5 | 6 | 7 | 8 | 9 |
| Sadness  (% duration) | .34  (3.62) | .23  (1.74) | 4.07  (14.63) | 8.81  (25.69) | 12.22  (29.14) | 9.15  (22.28) | 9.46  (27.32) | 12.35  (31.81) | 9.65  (25.69) |
| Interest-worry  (% duration) | 2.58  (13.73) | 6.34  (18.23) | 12.17  (27.36) | 12.91  (30.26) | 11.01  (27.57) | 9.62  (26.69) | 6.68  (21.53) | 10.13  (25.08) | 9.91  (24.31) |
