# Supplementary material for: Young Children’s Affective Responses to Another’s Distress: Dynamic and Physiological Features
Source: PLoS One. 2015 Apr 13;10(4):e0121735. doi: 10.1371/journal.pone.0121735 (PMC4395218; doi:10.1371/journal.pone.0121735)
Supplement: S1 Appendix — Detailed narrative of mother-infant separation vignette (study 1) and First Day vignette (study 2) by epoch (DOCX) [file pone.0121735.s003.docx]

S3 Appendix. Detailed narrative of separation vignettes

Detailed narrative of mother-infant separation vignette (study 1) and First Day vignette (study 2) by epoch

**Study 1: Mother-infant sepatation vignette** (narration in parentheses)

Epoch 1: Neutral

Mother and baby enter the surgery. (*This is a story about Tom and his mummy. They’ve gone to the dentist. Tom’s mummy needs to see the dentist)*

Epoch 2: Transitional

There is a knock at the door. (*Now Tom’s mummy can go and see the dentist. She goes into the room next door to see the dentist).* Mother leaves baby alone, (*Tom has to wait by himself while his mummy is with the dentist)*

Epoch 3: Separation

Baby is in the waiting room alone. He begins to cry.

**Study 2: First Day vignette (10 second epochs; final 3 seconds omitted)**

Epoch 1 (comparison period)

Young boy and his parents enter the school playground. Boy says he doesn’t want to go to school and shows some signs of protest Epoch 2

Epoch 2 (comparison period)

Boy runs back out of the school gates and his parents must go and bring him back

Epoch 3 (comparison period)

Boy is taken into his classroom by his parents, and becomes visibly distressed

Epoch 4 (critical period)

Parents attempt to leave and boy holds onto his mother and pleads with her to stay. Teacher restrains him

Epoch 5 (critical period)

Parents leave. Boy is visibly crying and yelling for his mother while the teacher tries to prevent him from running after his mother and father

Epoch 6 (critical period)

Boy, highly distressed, sobs and struggles against the teacher who is trying to contain his behavior and put him on her lap

Epoch 7

Boy, now sitting on teacher’s lap and clearly less resistant to comfort, starts to calm down and begins to take an interest in the teacher’s activity. There is a brief, clear expression of strong sadness prior to the transition to epoch 8

Epoch 8

At the start of the epoch, the boy is sitting on the teacher’s lap and his behavior is more contained, though he is clearly sad. The remaining episode in the epoch depicts the boy sitting with his peers at the end of the school day participating in a classroom activity

Epoch 9

Boy’s mother returns to pick him up, and he excitedly shows her his schoolwork
